# Bidirectional competitive interactions between motor memory and declarative memory during interleaved learning

**DOI:** 10.1038/s41598-020-64039-8

**Published:** 2020-04-23

**Authors:** Sungshin Kim

**Affiliations:** 1 Center for Neuroscience Imaging Research, Institute for Basic Science, Suwon, 16419 Republic of Korea; 2 Sungkyunkwan University, Suwon, 16419 Republic of Korea

**Keywords:** Learning and memory, Motor control

## Abstract

Distinct motor and declarative memory systems are widely thought to compete during memory consolidation and retrieval, yet the nature of their interactions during learning is less clear. Recent studies have suggested motor learning not only depend on implicit motor memory system supporting gradual tuning of responses by feedback but also depend on explicit declarative memory system. However, this competition has been identified when both systems are engaged in learning the same material (motor information), and so competition might be emphasized. We tested whether such competition also occurs when learning involved separate motor memory and declarative information presented distinctly but yet in close temporal proximity. We measured behavioral and brain-activity correlates of motor-declarative competition during learning using a novel task with interleaved motor-adaptation and declarative-learning demands. Despite unrelated motor versus declarative information and temporal segregation, motor learning interfered with declarative learning and declarative learning interfered with motor learning. This reciprocal competition was tightly coupled to corresponding reductions of fMRI activity in motor versus declarative learning systems. These findings suggest that distinct motor and declarative learning systems compete even when they are engaged by system-specific demands in close temporal proximity during memory formation.

## Introduction

Distinct neural systems supporting motor versus declarative memory^1^ are thought to operate simultaneously during motor learning, such as when one must adapt motor output to accommodate externally applied perturbations^2–6^. Declarative memory for goals, strategies, and feedback may dominate the earlier stage of learning, with gradual evolution of implicit motor memory that dominates later stages of learning and produces automaticity^7–11^. Thus, independent learning processes with distinct timescales operate simultaneously to achieve common learning goals, such as improving motor acuity, accuracy, and response times. Thus, interference between motor and declarative learning can also occur via competition while achieving the same goal for motor and declarative memory systems, probably due to limited memory resource shared by the systems^12^. For instance, in the consolidation period after motor learning, presentation of word-list material to be learned explicitly interferes with subsequent expression of the previous motor learning, suggesting motor-declarative competition during consolidation that may arise from consolidation bandwidth limits^12–16^. However, these studies retrospectively inferred interaction between the memory systems based on performance of tasks after consolidation period without any direct demonstration of underlying neural signatures^12,13,16^ or pre-assumed that specific brain regions (e.g., DLPFC, M1) are associated with separate memory processing^14,15^. Moreover, previous studies have shown competition either during learning for the common task goals or after learning for the separate system-specific task goals. However, it has been unclear whether the interactions would be competitive or cooperative during learning for the system-specific task goals. To fill this gap, we aimed to investigate direct evidences of the interaction between motor and declarative systems during learning by analyzing trial-by-trial performance of distinct motor versus declarative tasks.

**Figure 1:**
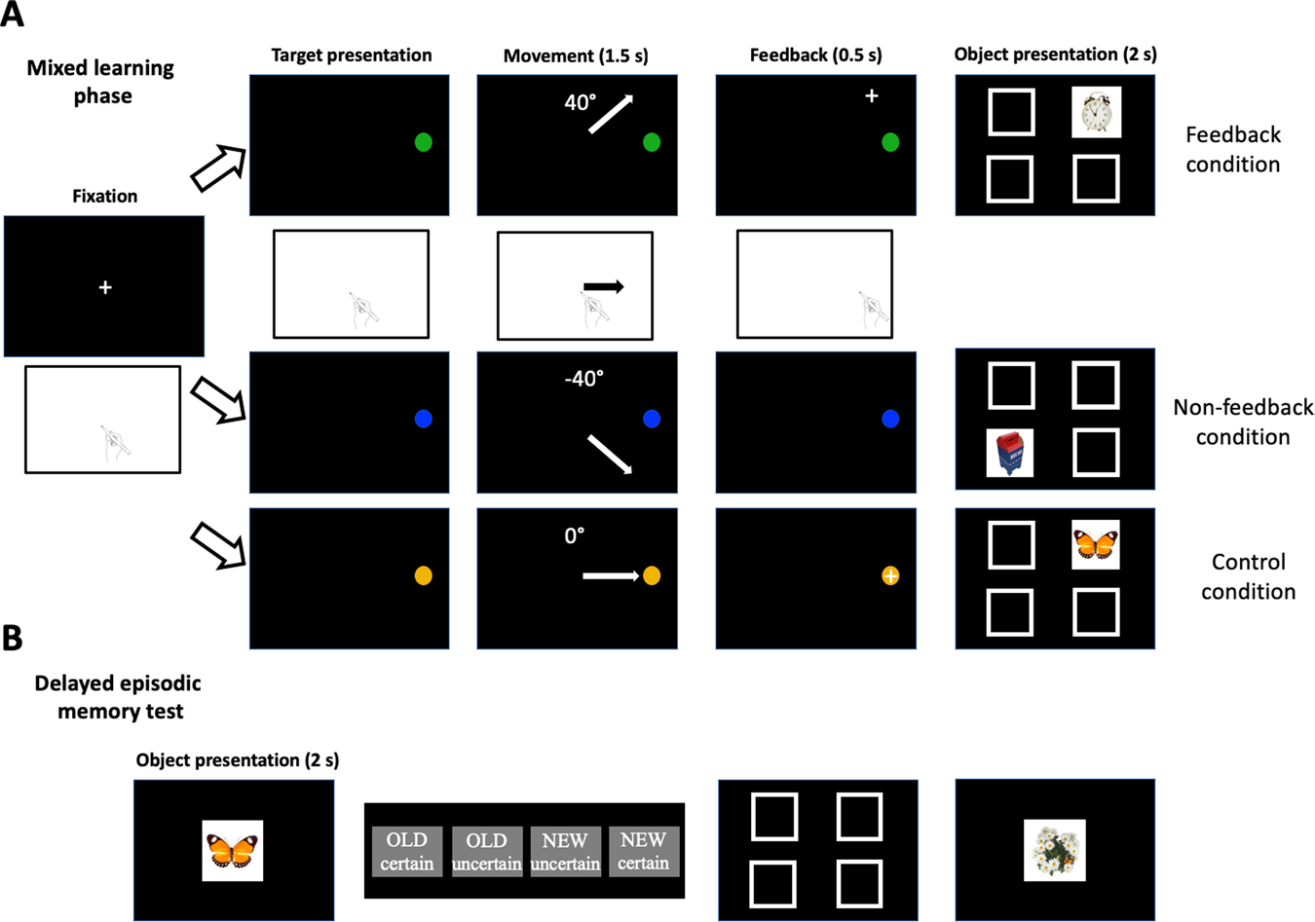
Experiment design. (**A**) For each trial in the interleaved learning phase, a target appeared on the right side of the screen and subjects attempted to reach the target using an fMRI-compatible pen on a tablet within 1.5 s (maximum movement time). Target colors cued different visuomotor rotations, 0°, +40°, and −40°. In the feedback and control conditions, a cursor position was provided as feedback for 0.5 s after the movement. Immediately after feedback, a trial-unique object appeared in the one of four locations. (**B**) In the delayed declarative memory test, we assessed object-recognition memory and recollection memory of the associated location. Object images were taken from an available open source, which was described in Brady *et al*.^63^, with no copyright protection via the internet for demonstration purposes.

Here, we developed a novel fMRI task in which subjects performed visuomotor adaptation, which is a standard type of motor learning^3–6,17^, interleaved with object-location association learning, which is a standard type of hippocampal-dependent declarative learning^18–21^ (Fig. 1). Visual feedback on which motor learning is dependent was either presented or hidden and trial-level motor error was used to quantify the amount of motor learning that occurred from one trial to the next. Additionally, we also designed a control motor task with feedback but without visuomotor adaptation to rule out possible effects of non-specific factors except for memory interference by the visual feedback *per se*, e.g., attention. Declarative memory testing after the learning phase was used to quantify the relative success of declarative learning on each trial with measuring recognition with the level of confidence and recollection of associated locations. This allowed us to quantify the degree to which motor and declarative learning occurred on a trial-by-trial basis. According to the hypothesis of competitive interaction between the memory systems, we predicted that motor learning would harm declarative learning and that the magnitude of this declarative memory disruption would scale with the magnitude of trial-by-trial motor learning. Likewise, we predicted that successful declarative learning would negatively affect the success of motor learning in temporally proximal trials.

To identify systems-level interactions supporting the predicted bidirectional interference, we fitted trial-by-trial measures of declarative learning and motor learning to fMRI activity. We predicted that activity of a motor-learning network including prefronto-parieto-cerebellar areas would positively correlate with the success of motor learning and would negatively correlate with the success of declarative learning. Likewise, we predicted that the negative influence of motor learning on declarative learning would correlate with reduced activity in an declarative-memory network including hippocampal and medial-prefrontal areas. This predicted pattern of findings would provide evidence for general competition between distinct motor and declarative memory systems; that is, competition even when learning involves distinct system-specific information presented in relatively close temporal proximity.

## Results

### Successful motor and declarative learning

**Figure 2:**
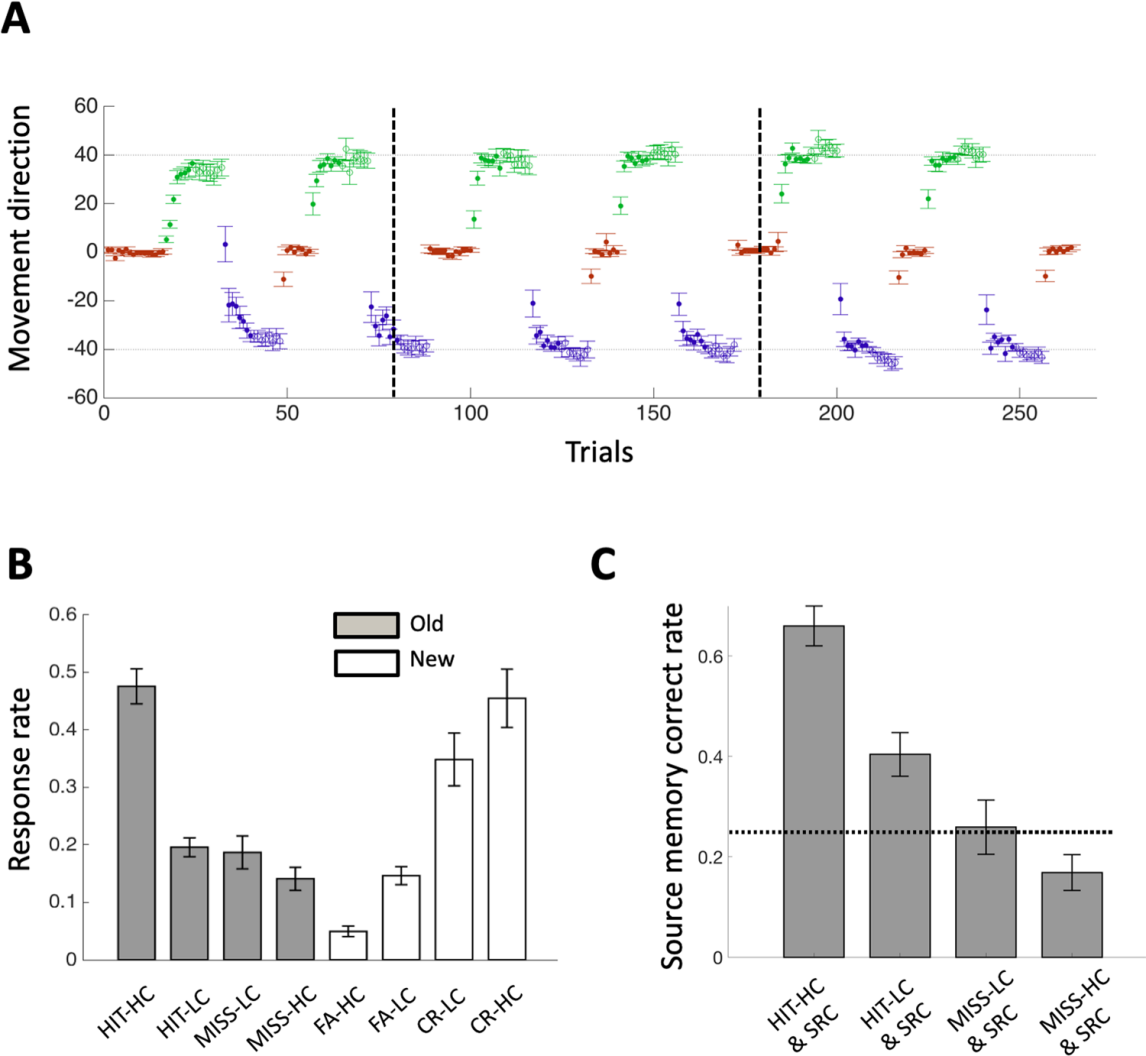
Motor and declarative learning. (**A**) Red, green, and blue circles indicate movement directions in Control (0° rotation), +40° Feedback, and −40° Feedback conditions, respectively, averaged across participants. The open circles indicate the non-feedback condition. (**B**) Responses rates for declarative recognition memory were averaged separately for old versus new objects and for each confidence level (HC: high confidence; LC: low confidence) and each response category (HIT, MISS for old objects, FA: false alarm, CR: correct rejection for new objects). (**C**) Correct response rates for source memory are shown averaged given the corresponding recognition response. The dotted line indicates the chance level of performance.

Motor learning was successful, as indicated by decreases in motor error within feedback blocks (for all blocks, 1-tailed t-test: *df* = 23, *p* < 0.0017, Cohen’s *d* = 0.69) that plateaued within non-feedback blocks (Fig. 2A). Performance worsened between the last trial of a feedback block and the first trial of the next feedback block of the same type due to forgetting, but increased overall across successive feedback and non-feedback blocks, as in prior studies^4,22^. In order to prevent premature learning and reliance on habitual responding, each subject successfully learned two distinct visuomotor rotations (+40 and −40 degrees)^4,22,23^. For each subject, errors in the last blocks for each rotation (feedback and non-feedback) were significantly lower than the imposed rotations (all *p* < 10^−7^). There was no difference in overall motor learning of the two rotations (*T*_(22)_ = 1.58, *p* = 0.13), and thus subsequent analysis collapsed these conditions. Control and non-feedback blocks were used as control conditions for which learning should have been minimal or absent. Indeed, there was no significant learning for most of control and non-feedback blocks (13 out of 16; *p* > 0.05, 1-tailed t-test) except for three non-feedback blocks in which marginal learning occurred (*p value range* = 0.022–0.048). Thus, motor learning was highly successful and based almost entirely on feedback.

Memory for object-location associations studied in an interleaved fashion during motor learning was assessed using a two-step test, whereby each trial was tested for recognition memory of the object followed immediately by recollection of its studied location. Object recognition was successful, as indicated by accurate discrimination of studied objects from new/unstudied objects (Fig. 2B). Subjects responded “old” with higher confidence to old objects and “new” with higher confidence to new objects (*F*_(3,66)_ = 96.4, *p* < 0.001, η_p_^2^ = 0.81 for the interaction of response type by object old/new status in a two-way repeated-measures ANOVA). All the subjects performed above chance, as indicated by discrimination sensitivity scores (*d*’) computed irrespective of confidence that were greater than zero (mean = 1.36, range = 0.30–2.59).

**Figure 3:**
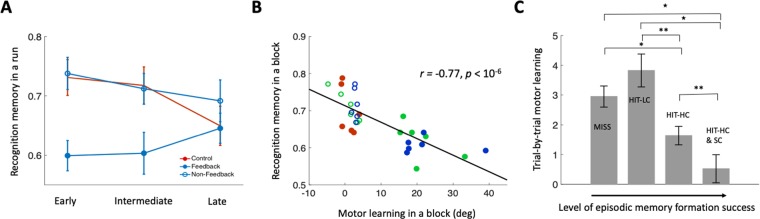
Reciprocal interference between motor and declarative learning. (**A**) Recognition memory performance is significantly lower in feedback blocks (in which motor learning occurred) than in the non-feedback and the control blocks that were devoid of motor learning and that controlled for various nonspecific factors (see text). (**B**) Motor and declarative learning levels correlated negatively across blocks, with conditions indicated via coloration as in Fig. 2A. (**C**) There was less motor learning (improvement from one trial to the next) with increasingly more successful declarative learning. (*p < 0.001, **p < 0.01, *p < 0.5).

Recall of object locations (i.e., source-memory recollection) was also successful and was strongly related to high-confidence object recognition^24^. To test whether the object recalls were from random guess or from true memory, we calculated the correct recall rates out of trials in which subjects chose to answer (i.e., clicking one of four location boxes). Object-location recall rates were significantly higher than the chance level of 0.25 (Fig. 2C) when subjects recognized objects with high confidence, (0.66 ± 0.040, mean ± SE; *T*_(22)_ = 10.28, *p* < 0.001, Cohen’s *d* = 2.14) and low confidence (0.40 ± 0.045; *T*_(21)_ = 3.45, *p* < 0.005, Cohen’s *d* = 0.74). However, they were not significantly higher than chance when subjects failed to recognize objects regardless of confidence (0.23 ± 0.049; *T*_(17)_ = 0.41, *p* = 0.69) (Fig. 2C). Because the recall rates were higher in more successful recognition memory from the above analyses, they can provide a graded declarative memory outcome when combined with recognition memory as in Fig. 3C. Object-location recalls were considered valid only when objects were successfully recognized. Thus, in the subsequent analysis, we assessed the source-memory recollection performance as the fraction of the locations recollected correctly out of all the recognized objects regardless of confidence (see details in Methods).

### Motor learning impaired declarative learning

To identify the influence of motor learning on declarative learning, a two-way repeated-measures ANOVA was performed on object-recognition memory performance and source-memory recollection performance, with subjects as random factors, and motor-feedback conditions (control, feedback, and non-feedback) and three experimental runs as repeated-measures. Declarative memory varied for the control, feedback, and non-feedback conditions irrespective of run (recognition: main effect *F*_(2,44)_ = 14.44, *p* < 0.001, η_p_^2^ = 0.40; recollection: main effect *F*_(2,44)_ = 3.51, *p* = 0.039, η_p_^2^ = 0.14), and this effect of feedback conditions varied across runs for recognition memory (interaction effect *F*_(4,88)_ = 4.30, *p* < 0.005, η_p_^2^ = 0.16), but not for recollection memory (interaction effect *F*_(4,88)_ = 0.73, *p* > 0.5). Post-hoc pairwise tests indicated that recognition memory and recollection memory were worse for objects learned in feedback blocks (when motor learning occurred) than in control blocks (recognition: *T*_(22)_ = 3.99, *p* < 0.001, Cohen’s *d* = 0.83; recollection: *T*_(22)_ = 2.25, *p* = 0.035, Cohen’s *d* = 0.47) and in non-feedback blocks (recognition: *T*_(22)_ = 5.29, *p* < 0.001, Cohen’s *d* = 1.10; recollection: *T*_(22)_ = 2.71, *p* = 0.013, Cohen’s *d* = 0.57), when no motor learning occurred (Figs. 3A and S1A). The overall impairment of declarative memory, which is measured as reduced performance in feedback blocks compared to other two conditions, was larger for the recognition memory than for the recollection memory (*T*_(22)_ = 2.12, *p* = 0.046, Cohen’s *d* = 0.44). Thus, in the subsequent analysis, we primary focus on the recognition memory unless otherwise stated (see supplementary Figures for recollection memory).

We tested whether there is any interaction between impaired declarative memory and motor responses, i.e., counteracting downward and upward reaching direction as responses to +40 and −40 degree visuomotor rotations. We hypothesized that movement responses could selectively interfere with subsequent declarative memory encoding for objects appeared in the movement directions. Alternatively, they serve as an additional contextual cue for object-locations, facilitating the declarative memory encoding. However, a two-way repeated-measures ANOVA found neither of significant main effect of object locations (recognition: *F*_(3,66)_ = 0.34, *p* = 0.80; recollection: *F*_(3,66)_ = 2.30, *p* = 0.085) nor significant interaction of rotation types by object locations on the impairment of memory scores (recognition: *F*_(3,66)_ = 0.61, *p* = 0.61; recollection: *F*_(3,66)_ = 0.58, *p* = 0.63). Unlike our expectations, the movement directions in the motor task neither selectively interfered with nor facilitated subsequent declarative memory encoding of object-locations.

Finally, we also tested whether nonspecific effect such as attention accounts for the difference in declarative memory between the conditions. Interestingly, reaction time varied for the three conditions, increasing in the order of control, feedback, and non-feedback conditions (*F*_(2,44)_ = 20.80, *p* < 0.001, η_p_^2^ = 0.49). Post-hoc pairwise tests indicated, in feedback blocks, the reaction time (703.1 ± 96.0 ms, mean ± SE) is longer than in control blocks (686.3 ± 85.5 ms) (*T*_(22)_ = 2.37, uncorrected *p* = 0.027), but shorter than in non-feedback blocks (730.6 ± 95.3 ms) (*T*_(22)_ = 4.30, uncorrected *p* < 0.001). However, when combining control and non-feedback conditions as a non-motor learning condition, reaction time is not different from that in a motor learning condition (feedback) (*T*_(22)_ = 0.94, *p* = 0.36). Thus, attention, which could be measured by reaction time, may not account for worse declarative memory in motor learning blocks. Furthermore, there was no significant difference in declarative memory for objects learned in control blocks versus non-feedback blocks (recognition: *T*_(22)_ = 0.74, *p* = 0.46, recollection: *T*_(22)_ = 0.02, *p* > 0.5) despite of large difference in reaction time between two conditions (*T*_(22)_ = 6.08, *p* < 0.001, Cohen’s *d* = 1.27), with performance for these conditions highly correlated across participants (recognition: *r* = 0.72, *p* < 0.001, recognition: *r* = 0.74, *p* < 0.001). In sum, motor learning that occurred during feedback blocks was associated with worse declarative learning, relative to non-feedback and control blocks. Non-feedback and control blocks were included to guard against nonspecific influences of motor and attentional demands on declarative memory^25–27^, and they were roughly matched in any influence they had on declarative memory.

To determine whether the amount of motor learning correlated negatively with the success of declarative learning, we computed the average motor learning within individual learning blocks and the success of declarative memory for the same blocks. These values were highly negatively correlated (*r* = −0.76, *p* < 0.001, Fig. 3B) for recognition memory and for recollection memory (*r* = −0.47, *p* = 0.011, Fig. S1B), indicating that the relative success of motor learning was highly related to the relative failure of declarative learning.

### Declarative learning impaired motor learning

Declarative memory formation impaired the motor learning that occurred on the trials following the object-location encoding. Trial-by-trial motor learning was calculated as the improvement (decrease of error) from the current to the next trial. We assessed this improvement value following object-location trials that were categorized into four increasing levels of declarative learning success based on subsequent performance (later-missed, later-hit with low-confidence, later-hit with high-confidence, and later-hit with high-confidence and source-correct). Motor improvement scores varied by declarative learning success (main effect *F*_(3,22)_ = 13.4, *p* < 0.001, η_p_^2^ = 0.38), with less improvement occurring for relatively more successful declarative learning (Fig. 3C). Post-hoc pairwise tests indicated less motor improvement for the highest level of declarative learning success compared to all lower levels (*p* < 0.008; Fig. 3C) and for the next-to-highest level of declarative learning success compared to all lower levels (*p* < 0.02; Fig. 3C), with no significant difference in motor improvement for the two lowest levels of declarative learning success (*T*_(22)_ = 1.32, *p* = 0.20). Thus, just as there was a negative impact of motor learning on the immediately forthcoming declarative learning trial, declarative learning disrupted the forthcoming motor learning.

### Network activity reflecting declarative learning impairment by motor learning

**Figure 4:**
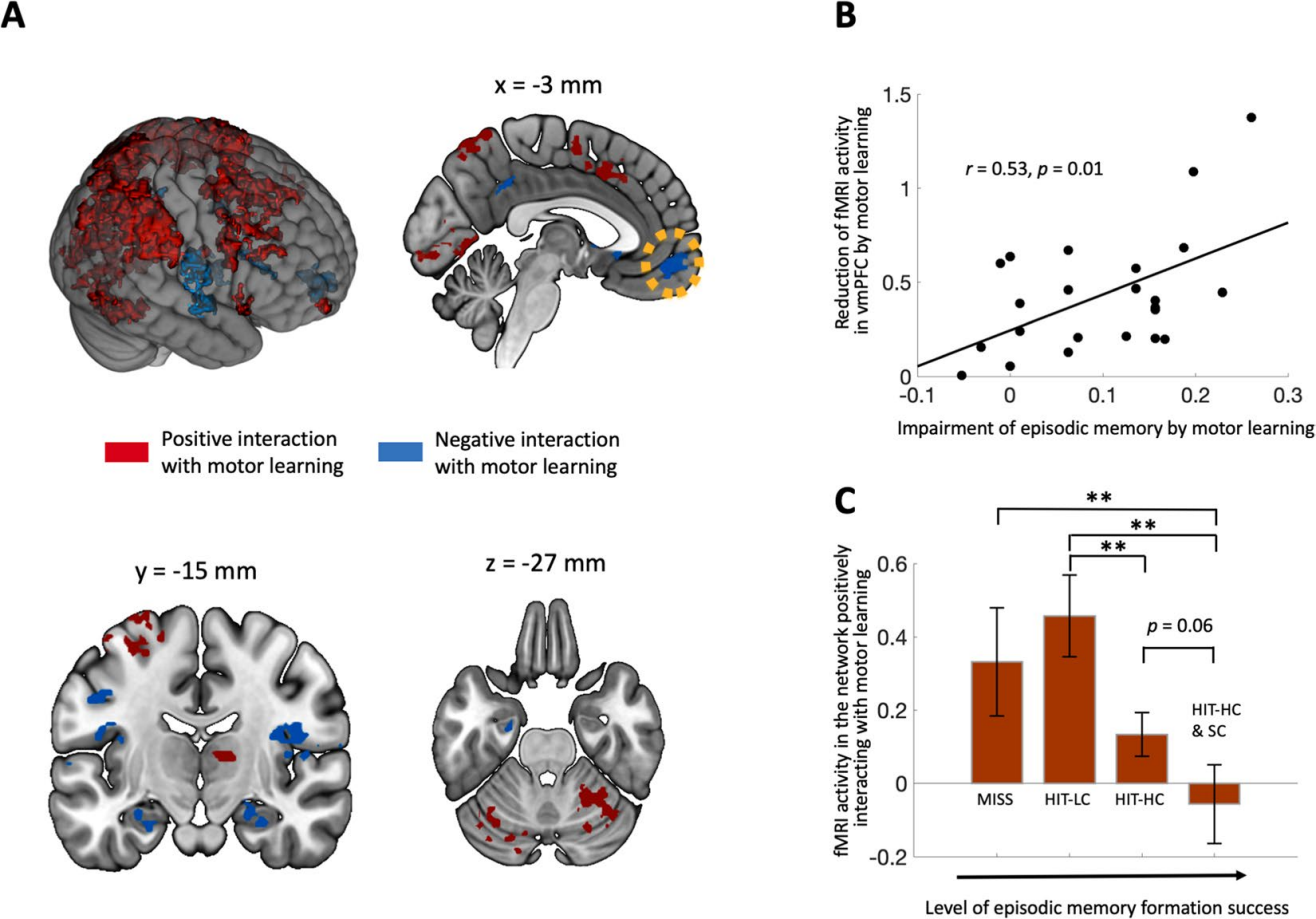
Reciprocal interference of brain activity reflecting motor and declarative learning. (**A)** Motor learning correlated positively (red) with activity of a prototypical prefronto-parieto-cerebellar motor-learning network and negatively (blue) with activity of a prototypical hippocampal-prefrontal declarative network. (**B**) The extent of negative modulation of fMRI activity within vmPFC, marked with a yellow-dotted circle in (A), predicted the level of declarative learning impairment by motor learning shown in Fig. 3A. (**C**) fMRI activity of the motor learning network (positive clusters in A) decreased with higher level of declarative memory formation success.

To identify brain activity reflecting the negative impact of motor learning on declarative learning, we first modeled fMRI activity reflecting trial-by-trial changes in motor performance error, which reflects the magnitude of motor learning^2–4,6,12,28–31^. Activity of a hippocampal-prefrontal network was negatively modulated by motor learning, such that activity in these areas, which are strongly associated with declarative memory^32–34^, was lower when more motor learning occurred on the immediately preceding trial (Fig. 4A, Table 1). Furthermore, subjects with greater levels of declarative learning impairment by motor learning (difference in recognition memory for objects studied during feedback versus non-feedback blocks) also had greater negative modulation of fMRI activity by motor learning within the vmPFC (*r* = 0.53, *p* = 0.01; Fig. 4B), which was the focus of the analysis as it was the location with the most robust negative modulation of fMRI activity by motor learning (Table 1).

**Table 1:** List of clusters significantly negatively interacting with motor errors.

	Peak (MNI)			Cluster size (voxels)	z-value at peak	Corrected *p*-value
	*x*	*y*	*z*			
*R Medial Frontal Gyrus*	9	55	−12	351	4.87	*p* < 0.01
*R Insula*	45	−13	19	257	4.69	*p* < 0.01
*L Hippocampus*	−25	−13	−24	154	4.96	*p* < 0.01
*R Superior Temporal Gyrus*	60	1	−2	98	5.14	*p* < 0.01
*L Superior Temporal Gyrus*	−67	−21	7	91	4.98	*p* < 0.01
*R Caudate*	1	18	−2	80	4.18	*p* < 0.01
*R Precentral Gyrus*	60	−1	14	74	4.79	*p* < 0.01
*R Hippocampus*	28	−15	−22	69	4.12	*p* < 0.01
*L Middle temporal Gyrus*	−59	−6	−22	45	4.88	*p* < 0.05
*L Postcentral Gyrus*	−47	−15	36	43	4.50	*p* < 0.05
	−42	−16	24	40	5.05	*p* < 0.05
*L Insula*	−35	−11	14	42	4.96	*p* < 0.05
*L Middle Cingulate Cortex*	−3	−47	36	39	4.49	*p* < 0.05

When we controlled out the potential effect of reaction time on the modulation given significant difference in reaction time between feedback and non-feedback blocks (*T*_(22)_ = 4.30, uncorrected *p* < 0.001, Cohen’s *d* = 0.90), the relationship remained significant (*r* = 0.54, *p* = 0.01). It was also significant when tested in the entire vmPFC region (Fig. S2A), which was defined by a meta-analysis of fMRI data repository (*r* = 0.44, *p* = 0.037, with controlling reaction time: *r* = 0.45, *p* = 0.035, Fig. S2B). These robust findings indicate that successful motor learning impaired subsequent declarative learning and decreased the associated fMRI signals in the declarative memory network.

### Network activity reflecting motor learning impairment by declarative learning

Activity within a prototypical prefronto-parieto-cerebellar network associated with motor learning^4,35–38^ was positively modulated by trial-by-trial motor performance error (Fig. 4A, Table 2). We tested whether increasing levels of declarative learning success was associated with subsequent reductions in fMRI correlates of motor learning within this network, which would mirror the effects identified on motor learning behavioral performance (Fig. 3C). Indeed, activity related to motor learning varied by the four levels of declarative learning success (main effect *F*_(3,22)_ =6.30, *p* < 0.001, η_p_^2^ = 0.22) (Fig. 4C), with post-hoc pairwise tests indicating that this was due to decreased activity with higher levels of declarative learning success (Fig. 4C), as was the case for the effects on behavior. This relationship was specific to the motor-learning network, with no similar relationship in the regions that negatively interacted with motor learning (*F*_(3,22)_ = 1.89, *p* = 0.14). Thus, successful declarative memory formation was associated with worse subsequent motor learning and reduced fMRI signals of motor learning in the prefronto-parieto-cerebellar motor network.

**Table 2:** List of clusters significantly positively interacting with motor errors.

	Peak (MNI)			Cluster size (voxels)	z-value at peak	Corrected *p*-value
	*x*	*y*	*z*			
*R Supramargnial Gyrus*	59	−28	46	5477	5.56	*p* < 0.01
*L Inferior Parietal Lobule*	−59	−35	48	3459	5.48	*p* < 0.01
*R Middle Frontal Gyrus*	40	24	41	856	5.05	*p* < 0.01
	35	65	−10	39	4.12	*p* < 0.05
*R Cuneus*	16	−79	9	789	4.89	*p* < 0.01
*R Middle Cingulate Cortex*	4	18	44	719	5.28	*p* < 0.01
*R Cerebellum (Lobule VI)*	23	−50	−29	633	5.12	*p* < 0.01
*R Superior Frontal Gyrus*	23	1	68	607	5.49	*p* < 0.01
*L Cerebellum (Lobule VI)*	−31	−66	−25	293	4.69	*p* < 0.01
*R Middle Occipital Gyrus*	40	−81	34	275	4.85	*p* < 0.01
*R Inferior Frontal Gyrus*	47	26	29	148	4.74	*p* < 0.01
	57	18	5	115	4.49	*p* < 0.01
*L Cerebellum (Crus 1)*	−16	−83	−27	112	4.71	*p* < 0.01
*R Middle Temporal Gyrus*	65	−57	−3	94	4.19	*p* < 0.01
*L Supplementary Motor Area*	−3	−4	60	79	4.98	*p* < 0.05
*L Superior Parietal Lobule*	−33	−52	70	52	4.28	*p* < 0.05
*R Thalamus*	11	−15	9	45	5.18	*p* < 0.05
*L Middle Frontal Gyrus*	−48	24	32	42	4.40	*p* < 0.05
	−42	35	32	38	3.82	*p* < 0.05

Finally, given that we found behavioral and fMRI evidences supporting bidirectional interference between motor and declarative learning networks, we expected to identify signatures of functional coupling between the learning networks from the PPI analyses. However, there were no regions in the whole brain showing significant PPI (i.e., modulation of functional connectivity between the networks by the degree of declarative or motor learning).

## Discussion

A novel fMRI experiment allowed measurement of trial-by-trial motor learning and the corresponding level of declarative memory formation success, providing behavioral and fMRI evidence supporting bidirectional interference between these systems. Previous findings have shown competition between motor versus declarative memory systems for common learning goals. The competition varies across different stages of motor learning, producing automaticity^7^ with decreasing dependence on declarative memory^11^. Motor and declarative memory systems also competitively interact during consolidation given distinct learning goals specific to each system^12–14^. The current results demonstrate these memory systems also compete during memory formation even when system-specific information is presented at distinct times. Thus, we provide another line of evidences supporting competitive interaction between two memory systems traditionally viewed as encapsulated and separated^1,39^.

Competitive interactions such as those identified here have also been seen for other types of learning systems. For instance, in reward-based learning involving competition between hippocampus and striatum^40,41^. However, like previous studies investigating motor-declarative competition during motor learning, studies of reward-based learning have typically observed hippocampal-striatal competition when subjects are tasked with learning one piece of information that could be relevant to both systems. Here, we segregated to-be-learned information into discrete yet temporally proximal packets, and nonetheless identified competition.

The experiment design intended to control non-specific effects such as attention and initial salience of the tasks by separating motor and declarative memory tasks in time, not simultaneous as typical studies employing dual-task learning paradigm^26,27,42,43^ and matching and controlling visual feedbacks and visuomotor rotations. The analysis on reaction time also supports the interference was less likely due to the non-specific effects. In addition, the interference remained significant in the intermediate and late stage of learning. However, the amount of interference significantly reduced across learning stages as the motor task probably became more automatic with less attentional demands. Thus, it is still not clear to what extent the divided attention by motor and declarative learning tasks affects the interference between them.

Movement directions in the motor task had neither interfering nor facilitating effects on subsequent object-location memory encoding. This null finding reflects the nature of interference between the motor and declarative memory could be more domain-general, not specific to visuospatial processing as we expected. Previous studies have shown a verbal declarative memory task such as word-list learning interferes with consolidation of motor memory both in motor adaptation^12^ and motor skill learning^13,14,44^. However, it remains unclear the verbal memory task also competes for memory resource with a motor task during memory encoding. Thus, in future studies, we could clearly elucidate the nature of interference by a series of new experiments with various declarative memory tasks.

The source of the competitive interaction is unclear and could be due to increased demands such as task switching^45–47^. Our robust finding that greater vmPFC deactivation was related to more interference suggest competition in some aspect of information integration functions supported by this region^48–51^. Most studies employing a dual-task paradigm aimed to understand interference presented in performing tasks *per se* by measuring reaction time and found overlapped neural representation between two tasks, supporting interference due to competition for the same brain region^25,52^. In contrast, our findings support separate neural substrates of different memory systems and attenuated activation in each system as a neural signature of the interference by the other memory processing although PPI analyses failed to identify direct functional coupling between memory systems. However, it is also possible that two learning systems are partially overlapped for common memory resource. Specifically, hippocampus processes not only declarative information but also motor information^53,54^ and cerebellum, in addition to its motor functions, is also engaged in a variety of cognitive functions including declarative memory processing^55–58^. For the future works, like a previous study^14^, brain stimulation targeting vmPFC with functional connectivity analysis could be considered to further investigate a potential role of the region in arbitrating motor-declarative competition.

One limitation of our study is that the design did not permit segregation of motor learning into fast/declarative versus slow/adaptation components^8,11,12^. Previous studies have identified declarative memory tasks interfered with motor learning during consolidation particularly for the fast/declarative component^12^. However, we have not tested this hypothesis in the current experiment. For this, we should measure delayed retention of motor learning, which is predictive of the slow component^59^, and design separate control trials without interfering declarative memory task (e.g., showing noise instead of objects) to test whether declarative learning affects the delayed retention of motor memory. For the other direction of interference, motor memory to declarative memory, either of fast or slow component seems to disrupt declarative memory given that the effect tended to decrease but remained significant in late stages of learning, when slow/adaptation components dominate performance. Nonetheless, stronger evidence could be obtained in future studies by segregating these components of motor learning and testing for their selective interaction with declarative learning.

Another potential limitation of our findings is that it is not fully clear whether the interference effects were anterograde versus retrograde. This is because motor and declarative learning events were interleaved and consecutive. Future studies could address this question by varying the time gaps between motor and declarative learning demands^17^ and/or by varying their order across trials.

In summary, our results provided behavioral and fMRI evidence supporting competitive reciprocal interaction between motor and declarative learning despite system-specific learning goals. Additional research is needed to further evaluate mechanisms for such competition and to test whether either of the systems is particularly dominant for producing such interaction, such as by selectively modulating one system versus the other using network-targeted brain stimulation^14,15,18,60,61^.

## Methods

### Participants

Behavioral and fMRI data were collected from twenty-five right-handed and neurologically healthy subjects (13 females, mean age = 25.7 years, age range = 19–35 years). Handedness was assessed by a modified version of the Edinburgh Handedness Inventory (score ≥ 50, for right-handedness^62^). All participants had normal or corrected-normal vision and were eligible for the experiment based on standard MRI safety screening. They gave written informed consent in accordance with the Declaration of Helsinki and were remunerated for their participation. The experimental protocol received approval from Northwestern University Institutional Review Board. Two subjects were excluded for no learning of motor tasks (one falling asleep in the scanner; see results for detailed exclusion criteria). Therefore, twenty-three subjects were included in data analysis (13 females, mean age = 25.7 years, age range = 19–35 years)

### Task procedures and experimental design

We designed an interleaved task-based fMRI experiment with motor learning and object-location association memory demands. On the beginning of each trial, a round target of 1 degree in visual angle appeared on the right of the screen, 5.8 degrees of visual angle away from the center. Subjects were instructed to manipulate an MRI-compatible tablet pen (Hybridmojo LLC, CA, USA) to move the cursor to the target within 1.5 s after the target onset (maximum movement time). Timed-out trials were considered invalid and excluded from data analysis (0.92% of all trials). There were five different experiment conditions; a control condition with visual feedback of the cursor movement, two visuomotor task conditions, in which the cursor movement was rotated 40**°** (counter-clockwise, Task 1) and −40**°** (clockwise, Task 2) with or without the visual feedback (Fig. 1). Three different target colors, yellow, blue, green, were used to differentiate the control task and two visuomotor tasks.

For the two visuomotor tasks with feedback, a cross-shaped white cursor (0.83 degree in visual angle) appeared as a current position (or rotated in visuomotor learning blocks) of the tip of the pen while movement, relatively from the center of the screen (shown as the initial position regardless of actual position in the tablet). Once the cursor crossed over 5.8 degrees of visual angle from the center, the cursor was fixed at the crossing point. The cursor was presented for additional 0.5 s after the maximum movement time. For the two visuomotor tasks with non-feedback, a cursor was not presented but subjects were instructed to move the pen to the target as if there was the cursor. With color codes of round targets, subjects could recognize a current task even without cursor feedback. Immediately after the round target disappeared, a trial-unique object (3.3 × 3.3 degrees of visual angle)^63^ appeared in one of four locations with pseudo-random order (permuting the four locations, Fig. 1) for two seconds. Subjects were instructed to remember objects with their appeared locations and move back to the home position. Object images were taken from stimulus sets developed for research purposes, which were made publicly available with no copyright protection via the internet (Computational Visual Cognition Lab; http://cvcl.mit.edu/MM; Cambridge, MA). Inter-stimulus-intervals (ISI: time during a fixation cross is presented between trials) were randomly generated from 2 s to 12 s from an exponential distribution, in 2 s increment.

The fMRI experiment was divided in three runs, lasting 700, 668, and 730 s, respectively with a short break (~30 s) between runs. Each experimental block consisting of eight trials and was presented according to schedules such as C11′22′C11′22′ (12 subjects) or C22′11′C22′11′ (11 subjects) per run, where C, 1, and 2 indicates a block of the Control, Task 1, and Task 2 and the dash mark indicates non-feedback block of the equivalent task. Thus, there were 30 experimental blocks in three runs, 6 blocks for each condition (Control, Tasks 1 and 2 with or without feedback), in terms of trials, 48 trials for control, 96 trials each for feedback and non-feedback conditions. Possible confounding effects due to schedule and target color were eliminated by counter-balancing two schedules and three color-codes across subjects. To avoid primacy and recency effects^64^, eight control task trials were added to the beginning and the end of the experiment, and four control task trials were added to the beginning of second of the third runs, constituting a total of 264 trials. These trials were not included main analyses except in Fig. 2A showing entire course of learning. Subjects practiced a familiarization session of ~100 trials of the control task before the experiment. Learning two opposing visuomotor rotations while performing the associative memory task takes hundreds of trials, thereby providing enough statistical power in fMRI analysis as well as making more likely interaction between motor memory and declarative memory.

In a delayed (~10 minutes) memory test session outside of a scanner, 528 objects were presented, half of which were old (presented in the fMRI experiment) and the other half new, in randomized order. On each trial, the object was presented in the center of computer screen for two seconds and a prompt appeared asking subjects to classify the object as old or new, each with two confidence levels, “certain” or “uncertain”. For old objects only, subjects were then asked to click one of four appeared boxes corresponding remembered location or click anywhere outside of four boxes if they do not remember. There was no time limit for these responses and two-second interval with the fixation-cross separated the response period from the next trial. The test session typically lasted approximately 45 minutes depending on responses of subjects.

Stimuli were presented on a computer screen and reflected onto a mirror mounted to the head coil. Subjects laid in a supine position in the scanner and had a tablet fixed on their lap so that they could reach all the corners of the tablet comfortably with their right hand.

### Behavioral data analysis

For each of 264 trials, we calculated the average and variability of movement direction across subjects (Fig. 2A). The angular error was calculated as a size of deviation between the target direction and the final cursor direction from the center of the screen. Trial-by-trial movement direction were averaged across subjects and displayed with error bars (SEM) to show overall performance of motor learning.

Recognition memory performance as fraction of later-hit trials out of 240 trials (30 blocks × 8 trials) excluding control trials in primacy and recency blocks (Fig. 2B). To test whether it is significantly above the chance level, 0.25 (Fig. 2C), source-recollection memory performance was first calculated for each of four recognition response types as fraction of correct responses out of responses with clicking one of four locations. Note that, for five subjects, there were no valid trials used to calculate source-memory recollection scores when they failed to recognize objects because they responded as “don’t remember” for all the trials by clicking outside of four response boxes. We found the source-memory recollection was not significantly different from the chance when the objects were not correctly recognized (see Fig. 2C). Thus, in the subsequent analysis, the source-recollection memory was assessed as fraction of responses with correct recollection of the associated locations out of correct recognition responses. Here, trials with “don’t remember” were also included to avoid overestimating the actual performance.

The declarative memory (object-recognition memory, source-recollection memory) performance was compared among three conditions, the control, feedback, and non-feedback across six experimental blocks (two for each run) and post-hoc paired t-tests were performed. We hypothesized the declarative memory performance in the feedback condition would be lower than those in the control and the non-feedback condition due to interference of motor learning (i.e., motor memory update) with declarative learning. Within each of 30 experiment blocks except for additional blocks of primacy and recency effects, we also estimated motor learning and related with the declarative memory performance. For each of 23 subjects, movement directions within each block were fitted by an exponential function with three free parameters (*A*, *B*, *C*) such that $x(t)=A\cdot \exp (-B(t-1))+C$, where *x*(*t*) is a movement direction at trial *t*, (*t* = 1, 2, …, 8). For robust estimation of learning with few trials, we excluded missed trials and trials with large (>20**°**) overshoot (less than 3.5%). Learning within a block was estimated as the amount of movement change on the fitted curve from the initial to the final trial. Then, the estimated amounts of learning within a block were averaged across subjects and correlated with averaged declarative memory performance.

We also investigated on the interference of the opposite direction, that is, from declarative learning to motor learning. For this, the 240 studied objects except for those used for primacy and recency effects (24 objects) were back-sorted to be categorized into four different levels of declarative memory formation success according to their responses in a delayed test session; later-miss (MISS: Level 1), later-hit with low-confidence (HIT-LC: Level 2), later-hit with high confidence (HIT-HC: Level 3), later-hit with high-confidence and source-correct (HIT-HC & SC: Level 4). Then, we compared the trial-by-trial motor learning associated with the object, which was defined as the change of errors in motor learning task from the current to the next trials. Here, we hypothesized higher declarative memory formation success for a given object would more interfere with motor learning.

### MRI data collection and preprocessing

MRI data were acquired using a 64-channel head/neck coil on a 3-tesla Siemens TIM Prisma whole-body scanner at Northwestern University Center for Translational Imaging.

A high-resolution T1-weighted structural 3D MP-RAGE was acquired before the task to provide anatomical location (voxel size: 1mm^3^; field of view: 256 mm, 151 sagittal slices). Whole-brain functional images were acquired during the fMRI experiment, 350, 334, and 365 scans for three runs, respectively. Scanning parameters were 2000-ms repetition time (TR), 20-ms echo time (TE), 210 mm field of view (FOV), 80° flip angle (FA), 1.7 × 1.7 × 1.7 mm isotropic voxels, and multi band factor of 2. Processing of fMRI data used freely available AFNI software^65^. All functional images were first corrected multi-band slice-timing (3dTshift), and then realigned to adjust for motion-related artifacts (3dvolreg). The structural image was skull-stripped (3dSkullStrip) and coregistered with functional images using normalized mutual information as a cost function (align_epi_anat.py, with option, “-nmi”). The realigned images were then spatially normalized with the MNI152 T1 template (@auto_tlrc). All functional images were smoothed using a Gaussian kernel of 4 × 4 × 4 mm full width at half maximum (3dmerge) and scaled to mean of 100 for each run (3dcalc).

### fMRI data analysis

We performed two general linear model (GLM) analyses on fMRI data to identify activity supporting bidirectional interference shown in behavioral results (see Results) and psychophyisiological interaction (PPI) analyses^66^ to identify functional coupling between motor and declarative memory networks. First, for the interference from motor learning to declarative learning, we designed a parametric regressor. Specifically, a boxcar function with amplitude modulated by trial-by-trial motor error, onset and duration encoding 2s-long presentation of object stimuli was convolved with a canonical hemodynamic. Here, we made important assumption that motor learning is proportional to motor error in the visuomotor adaptation task, as has been supported by previous studies^2–4,6,12,28–31^. For regressors of non-interest, we added boxcar functions encoding blocks of primacy and recency trials, six rigid-body motion regressors and six regressors for each run modeling up to 5th order polynomial trends in the fMRI time series. A beta-value of the error-modulating regressor was estimated via a general linear model incorporating hemodynamic response deconvolution (3dDeconvolve).

From the first GLM analysis, we defined two distinct networks, which were constructed by clusters of significantly positive and negative beta-values of the error-modulating regressors. For multiple comparison correction, voxel-wise threshold was set to *p* < 0.001 two-tailed and a Monte Carlo simulation determined 38 contiguous supra-threshold voxels (187 mm^3^) was needed to achieve cluster-wise corrected threshold *p* < 0.05 within the whole-brain group mask (3dttest++ with the option “-Clustsim”)^67^. Each of positive and negative interaction networks consisting of the significant clusters was overlaid over the MNI template brain. The visualization for the two networks (Fig. 4A) was performed using open-source 3-dimensional rendering software, MRIcroGL (McCausland Center for Brain Imaging, University of South Carolina, http://www.mccauslandcenter.sc.edu/mricrogl/).

Then, we sought to test whether the deactivation of the hippocampal-prefrontal memory network negatively interacting with motor learning, is correlated with the extent of the interference with declarative memory formation due to motor learning. For this, the averaged beta-values of the error-modulating regressor in the vmPFC (the most robust cluster identified by the primary fMRI analysis) were correlated with the declarative memory accuracy reduction in feedback blocks compared to following non-feedback blocks across subjects (see Fig. 3A). It is notable that trial-by-trial motor errors used for the parametric regressor are inherently independent measures from the individual levels of interference with declarative memory due to motor learning^68^. These two independent behavioral measures turned out to be negatively correlated (Fig. 3A,B) and this is the main finding of the current study. The fMRI analysis with selection of the region of interest (vmPFC) based on the error-modulating regressor would not bias the regression analysis for the independent behavioral scores. However, we further tested the results with the entire vmPFC region defined by a term-based meta-analysis using a key word, “ventromedial prefrontal” from neurosynth database, which retrieved 333 studies and 12062 activations (see supplementary Fig. S2A, rendered using MRIcroGL software).

Second, for the interference from declarative memory formation to motor learning, we generated four regressors encoding the onsets of motor task feedback only in the trials of the feedback blocks, which were classified depending on a response in a delayed test session; ‘hit with high confidence with source-memory correct’, ‘hit with high confidence with source-memory incorrect’, ‘hit with low confidence’, and ‘miss’. Each regressor consisted of eight stick regressors starting 1 s to 15 s after the target onset of motor task every 2 seconds aligned to TR. Experiment blocks of recency & primacy, control and non-feedback condition were separately modeled as regressors of non-interest. The other regressors of non-interest modeling head motion and polynomial trend were the same as those of the first regression analysis.

In the second regression analysis, we searched neural correlates of the interference for the opposite direction, from declarative memory formation to motor learning. To this end, the beta-values within the positive-interaction network were averaged for each of four regressors of the second regression analysis corresponding to a varying level of declarative memory success. To compare with behavioral results, we calculated the mean of two estimated beta-values of regressors, for “hit with high confidence” regardless of source memory response. Likewise the first regression analysis, selection of the region of interest (ROI) here (positive-interaction network) was based on the error-modulating regressor, which is inherently independent from declarative memory scores. Moreover, the fMRI activities measured as the beta-values of four separate regressors were also independent from those used to define the ROI (beta-values of the error-modulating regressor). Thus, this selective analysis is based on two independent statistics, without any double dipping issues.

Last, in the PPI analyses, we first generated the physiological regressors as timeseries extracted from each of the three clusters in the hippocampal-prefrontal memory network defined by the first regression analysis, vmPFC and bilateral hippocampus (see Table 1). For the psychological factors, we used three contrasts of hit versus miss, hit with high confidence versus everything else and among three conditions of the motor task, control, feedback, and non-feedback. For each of nine PPI analyses, which are combinations of three physiological regressors from the seed regions and three psychological regressors, we also included regressors encoding the onsets of a target separately for the psychological factors (psychological regressors), as well as a physiological regressor, in addition to the interaction regressor between physiological and psychological factors.

## Supplementary information

Supplementary information.

## Data Availability

All MRI data used in this study are archived in the Northwestern University Neuroimaging Data Archive (NUNDA, https://nunda.northwestern.edu) and can be downloaded following registration. The corresponding author can provide any information on dataset identification within the NUNDA system if necessary. Additional data related to this paper may be requested from the corresponding author.
