# Supplementary material for: Bidirectional competitive interactions between motor memory and declarative memory during interleaved learning
Source: Sci Rep. 2020 Apr 23;10:6916. doi: 10.1038/s41598-020-64039-8 (PMC7181727; doi:10.1038/s41598-020-64039-8)
Supplement: Supplementary file 1 — Supplementary information. [file 41598_2020_64039_MOESM1_ESM.docx]

**Bidirectional competitive interactions between motor memory and declarative memory during interleaved learning :**

**Supplementary Material**

Sungshin Kim^1,2,3^

^1^Center for Neuroscience Imaging Research

Institute for Basic Science

Suwon 16419, Republic of Korea

^2^Sungkyunkwan University

Suwon 16419, Republic of Korea

^3^Department of Medical Social Sciences,

Northwestern University

Chicago, IL 60611, USA


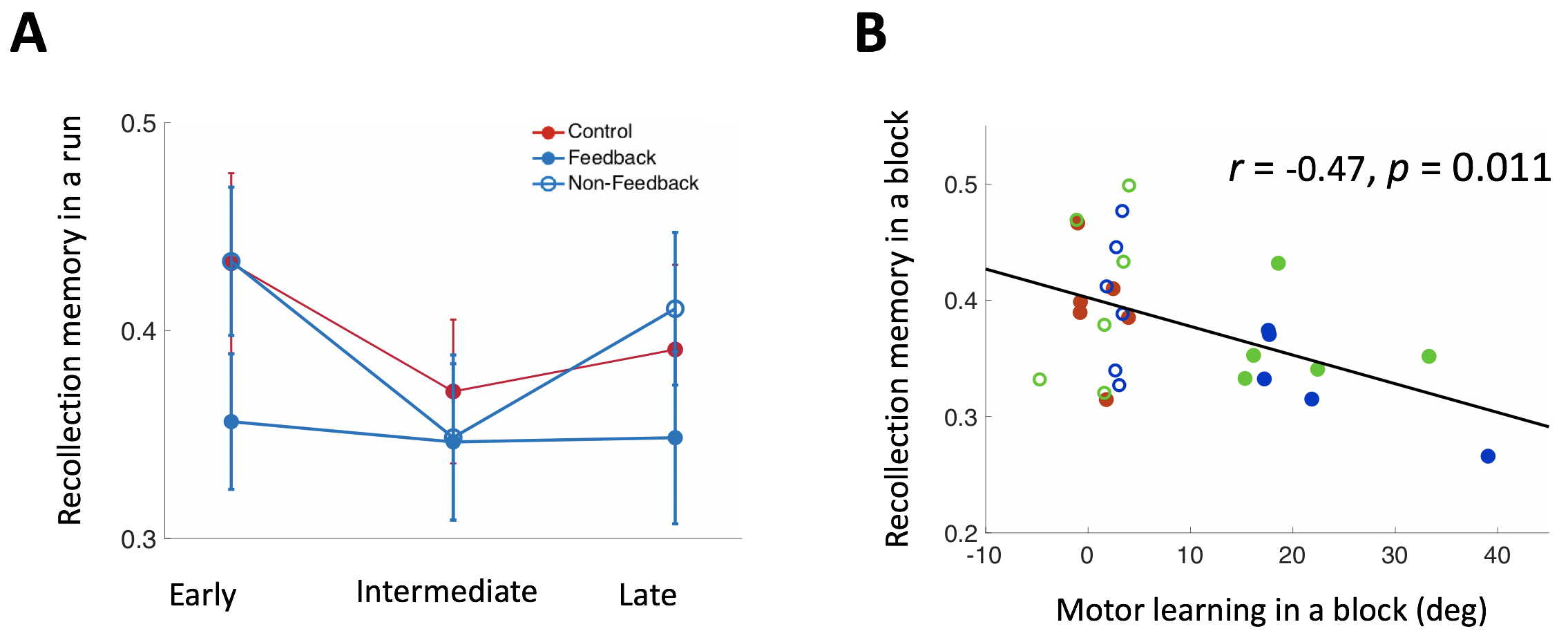


**Figure S1. Interference from motor learning to source-recollection memory** (**A**) The interference effect (reduction of memory performance in motor learning condition, feedback) was significantly lower for object-location recollection memory than for object-recognition memory (Figure 3A) (**B**) Recollection memory and motor learning levels correlated negatively across blocks, but showed weaker effect than the case for recognition memory (Figure 3B).


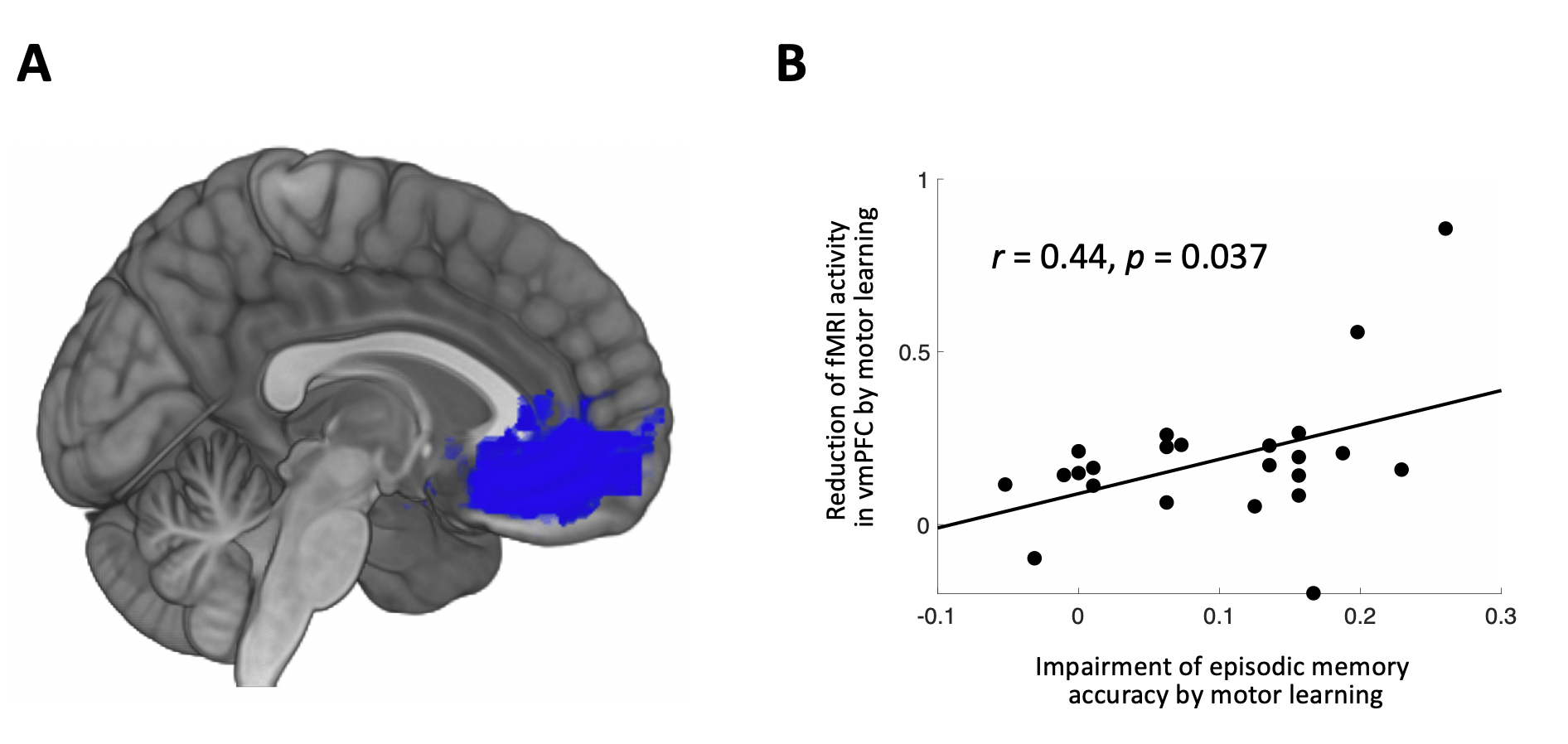
**Figure S2. fMRI correlates of interference from motor learning to declarative learning (A)** Entire ventromedial prefrontal cortex as a region of interest defined as a term-based meta-analysis using a key word, “ventromedial prefrontal” from neurosynth database, which retrieved 333 studies and 12062 activations **(B)** Larger deactivation in vmPFC was related with larger impairment of declarative memory by motor learning, supplementary to Figure 4B.
